# The impact of homocysteine, B_12_, and D vitamins levels on functional neurocognitive performance in HIV-positive subjects

**DOI:** 10.1186/s12879-019-3742-8

**Published:** 2019-02-04

**Authors:** Katia Falasca, Marta Di Nicola, Giuseppe Di Martino, Claudio Ucciferri, Francesca Vignale, Alessandro Occhionero, Jacopo Vecchiet

**Affiliations:** 1 Clinic of Infectious Diseases – Department of Medicine and Science of Aging, University “G. d’Annunzio” Chieti-Pescara, Pescara, Italy; 2 Laboratory of Biostatistics, Department of Medical, Oral and Biotechnological Sciences, University “G. d’Annunzio” Chieti- Pescara, Pescara, Italy; 3 School of Hygiene and Preventive Medicine - Department of Medicine and Science of Aging, University “G. d’Annunzio” Chieti-Pescara, Pescara, Italy; 4 Department of Medicine and Health Sciences, University of Molise, Campobasso, Italy

**Keywords:** Sulphur amino acids, Vitamin, Neuropsychological examination, Cognitive impairment

## Abstract

**Background:**

The correlation among high levels of total homocysteine, low levels of B_12_vitamin, and neurocognitive impairment in HIV negative patients has been the main research topic in some of the latest reviews. The aim of this study was to examine if the alteration of homocysteine, B_12_ vitamin, and D vitamins plasma levels was present in HIV-positive, and their relationship with cognitive function.

**Methods:**

57 HIV infected were enrolled and underwent the serum measurement of homocysteine, B_12_, and D vitamins. The neurocognitive evaluation investigated 5 cognitive domains, through a neuropsychological battery test

**Results:**

Homocysteine was found to be elevated in 70.2% of cases, B_12_ vitamin mean levels were low in 8 participants (14.0%), and 8 patients had D hypovitaminosis (14.0%). Abnormal homocysteine levels were associated with worse performance of verbal fluency (*p* = 0.003) and worse executive function (Stroop E test *p* = 0.040). The 25-OH D hypovitaminosis was associated with worse performances in executive functions in three different tests: Stroop E (*p* = 0.049), Trail B (*p* = 0.035), and Wais Digit Span (*p* = 0.042). Pathological levels of B_12_ Vitamin were also associated to worse performances in executive functions (Trail B Test and Wais Digit Span respectively *p* = 0.002 and 0.029) and with a lower speed in psychomotor processing (Peg Board Test on dominant hand, *p* = 0.014).

**Conclusions:**

In this study serum homocysteine, B_12_, and D vitamin levels are associated with neurocognitive performances; in fact low performance neurocognitive was correlated with hyperhomocysteine and low B_12_vitamin, and D vitamin levels. Evidence of the alteration of these parameters could facilitate the early identification of a neurocognitive impairment*.*

**There are no conflict of interest. No financial support was given for the study.**

## background

The prevalence of HIV-associated neurocognitive disorders (HAND), especially asymptomatic and milder forms, remains high even in patients undergoing a stable and successful combination antiretroviral therapy (cART) [1]. The presence of risk factors for HAND should heighten clinical suspicion for the disorder; these include host factors, HIV disease factors, and comorbidities. Host factors include genetic predisposition and metabolic disorders. More important are the associations of HAND or ANI (Asymptomatic Neurocognitive Impairment) with metabolic disorders, aging, and vascular disease [2].

The metabolism of homocysteine depends on folate, B_12_, and B_6_ vitamins. Therefore elevated levels of homocysteine are a marker of B_12_ and/or folate deficiency [3]. In a longitudinal HIV-negative population based study of older adults without dementia, higher levels of B_12_ vitamin and lower levels of homocysteine were associated with a decreased rate of loss brain volume over 6 years [4]. Rates of brain atrophy were significantly correlated with levels of homocysteine in the Study on Cognition and Prognosis in the Elderly [5], and lower levels of B_12_ vitamin were associated with an increased rate of brain volume loss in the Oxford Project to Investigate Memory and Aging study [6]. B_12_ vitamin and folate have a strong connection with the metabolism of homocysteine, a sulphur-containing non-essential amino acid. High levels of homocysteine and low levels of B_12_ vitamin and folate are common in the elderly and correlate with several conditions, such as cardiovascular and cerebrovascular diseases [7]. These biochemical alterations could even have an impact on brain structure through several physiopathological pathways [8]. In older HIV-negative adults, higher blood levels of homocysteine have also been associated with brain atrophy [9] and with increase of white matter lesions volume over time [10], although more studies are needed to confirm these data [11].

D vitamin deficiency has been associated with a proinflammatory state that induces blood vessels injury but is also connected to other vascular risk factors [12], and perhaps these vascular disorders cause neurocognitive disorders.

However, to our best knowledge, very few studies have examined to the association of homocysteine with brain damage, evaluated by neurocognitive deficit, in subjects with HIV infection [13].

The aim of this study was to assess the prevalence of hyperhomocisteinemia in HIV+ patients and to examine the relationship between homocysteine, B_12_, and D vitamins levels and cognitive function evaluated through the analysis of five cognitive domains, which may represent useful peripheral indicators of brain activity.

## Methods

### Study design

Analytical transversal study including fifty-seven (57) caucasian participants with HIV infection who were under continuous ART, followed at the Clinic of the Infectious Diseases, Department of Medicine and Science of Ageing, “G. d’Annunzio” University (Chieti-Pescara, Italy) was performed.

Patients were clinically stable and virologically suppressed and had a CD4+ cell count > 300 cells/mL during the six months before the beginning of the observational period. The patients had no opportunistic infections during this time and no antiretroviral therapy changes in the year before the study started.

After that laboratotory and neurocognitive tests were performed, the patients received a D vitamin supplementation if they showed D hypovitaminosis, in accordance with the current guidelines.

During their baseline visit, blood sample was taken for routine laboratory analysis. Moreover, a clinical assessment was performed. Patients were advised to report any adverse events or changes in their condition throughout the study and to continue their usual diet and lifestyle. Excluding criteria were: (a) steroids, growth hormone, testosterone or any anabolic agent use in the previous six months; (b) drug and alcohol abuse; (c) acute infection in the previous three months; (d) kidney disease and reduced glomerular filtration rate; (e) acute hepatic disease and (f) previous diagnosis of HAND.

This study was conducted in accordance with the guidelines proposed in the Declaration of Helsinki. All participants gave informed consent for the study and the study was approved by the Ethics Committee at the University “G. d’Annunzio” Chieti-Pescara (Ethics Committee Project No.3 the 06/02/2012) and was performed in accordance with the ethical standards laid down in the 1964 Declaration of Helsinki. Overnight fasting venous blood samples were collected for immunological analyses during the intervention period for analysis.

### Neuropsychological examination

All patients underwent a comprehensive neurocognitive test evaluating memory, attention and working memory, executive functions, speed of psychomotor processing, and language. [14].

The following tests were used to examine the abovementioned neurocognitive skills. For memory: Immediate and Delayed recall of Rey’s words, Delayed recall of Rey’s figure; speed of psychomotor processing was evaluated through Grooved Pegboard Test for both dominant and non dominant hand, attention and working memory through Digit span Forward, Spatial span Forward, Double Barrage, executive functions though Stroop test, Trail Making Test B, Drawings, and WAIS Digit Symbol and language through Letter fluency. The Zung Depression scale in order to assess a mood disorder and the Instrumental Activities of Daily Living (IADL) Scale were also administered.

A trained medical doctor (KF) administered and scored all tests, adjusting them for age, gender, and education on the basis of data available for the general Italian population.

A mild cognitive impairment was diagnosed with a score lower than the normative cut-off in more than three tests [15].

### Biochemical analyses

We collected fasting venous blood samples from the antecubital vein of patients at their first examination, determining complete blood count, creatinine, plasma levels of triglycerides, total cholesterol, high-density lipoprotein (HDL)-cholesterol, low-density lipoprotein (LDL)-cholesterol, glucose, and albumin. Laboratory tests were performed at our hospital in the Division of Clinical Pathology.

Serum D vitamin concentrations were measured in EDTA by radioimmunoassay, DiaSorin, Balton -UK – according to the manufacturer’s instructions. B_12_ plasma vitamin and serum folate were quantified using an electrochemiluminescence immunoassay on a cobas e analyzer (Roche, Penzberg, Germany). Total homocysteine was measured in plasma samples by stable isotope dilution liquid chromatography tandem mass spectrometry using a Quattro micro instrument (Waters Corporation, Milford, MA, USA), as described by Magera et al. [16]

### Virologic and immunologic markers

Plasma viral load (HIV-RNA) was determined using the “Amplicor” method (Roche Molecular Diagnostics, Milan, Italy) with a sensitivity of 40 HIV RNA copies/mL of plasma. CD4 + − and CD8 + −T cell counts were obtained by flow cytometry of lymphocyte subpopulations.

### Statistical methods

According to the main endpoint of the study, the sample size determination was based on the prevalence of hyperhomocysteinemia in HIV population based on previously observed data and published results [17]. Assuming a prevalence of 70%, with a precision of 10% and a 95% confidence interval, at least 57 patients were needed. Quantitative variables were summarized as mean and standard deviation (SD) or median and interquartile range (IQR) according to their distribution; qualitative variables were summarized as frequency and percentage. A Shapiro-Wilk’s test was performed to evaluate the departures from normality distribution for each variable. Mann-Whitney U-test was performed to evaluate differences of Homocysteine, B_12_, and D vitamins between patients with and without pathological value of cognitive test. Logistic regression models were performed to estimate the relationship between Homocysteine, B_12_, and D vitamins and ANI diagnosis. The results of models were expressed as odds ratio (OR) and relative 95% confidence interval (95% CI). The number of type I errors, was controlled applying the Gavrilov-Benjamini-Sarkar procedure to bound the false discovery rate (FDR) ≤0.05. All statistical tests were evaluated at an alpha level of 0.05. Statistical analysis were performed using IBM® SPSS Statistics v 20.0 software (SPSS Inc., Chicago, Illinois, USA).

## Results

### Study population

A total of 60 Italian participants (50 males, 83.3%), with 51.0 ± 9.8 years of age and 10.8 ± 3.1 years of mean education were enrolled. Three participants were not included into analyses due to incomplete serum sampling. All patients were in cART without therapeutic changes for more than 12 months. All patients were on cART according to currently accepted guidelines. Viral load was persistently undetectable and the median CD4+ cell count was 646 cells/μl (IQR 523–964 cells/μl). The mean serum concentrations of main metabolic values, such as glucidic and lipidic parameters, showed no meaningful discrepancies. Markers of liver and kidney functions did not show any substantial change compared to a range of normality. Furthermore, the mean BMI (Body mass index) was 25.7 ± 3.6 kg/m^2^ (Table 1).

**Table 1:** Patient’s characteristics (*n* = 57)

Variable	
Age (ys)	51.0 ± 9.8
Gender, n(%)	
Male	50 (87.7)
Female	7 (12.3)
BMI (Kg/m^2^)	25.7 ± 3.6
Level of instruction (ys)	10.8 ± 3.1
History of HIV infection (ys)	11.1 ± 7.5
Risk factors, n(%)	
MSM	22 (38.6)
Ex-IDU	11 (19.1)
Heterosexual	24 (42.1)
Therapy, n(%)	
NRTI	53 (93.0)
NNRTI	20 (35.1)
IP	20 (35.1)
INSTIs	20 (35.1)
HCV, n(%)	9 (15.8)
HBV, n(%)	6 (10.5)
Glycaemia (mg/dl)	98.8 ± 48.8
Total cholesterol (mg/dl)	198.2 ± 44.2
HDL cholesterol (mg/dl)	51.8 ± 16.9
LDL cholesterol (mg/dl)	113.4 ± 40.0
Triglycerides (mg/dl)	164.8 ± 99.0
Creatinine (mg/dl)	1.0 ± 0.9
Albumin (g/dl)	4.6 ± 1.0
25-0H D Vitamin (ng/ml)	26.8 ± 9.3
Homocysteine (μmol/l)	15.1 ± 6.3
B_12_ Vitamin (ng/ml)	378.5 ± 151.8
Folate (ng/ml)	9.8 ± 13.7

*MSM* men sex with men; IDU injection drug users; *NRTI* nucleoside reverse transcriptase inhibitor; *NNRTI* non-nucleoside reverse transcriptase inhibitor; *IP* Protease Inhibitors; *INSTIs* Integrase strand transfer inhibitors

### *Homocysteine, B*_12_, *and D Vitamins*

The mean value of homocysteine was 15.1 ± 6.3 μmol/l and high levels of homocysteine (plasma level ≥ 12 μmol/l) [18] were found in 40 participants (70.2% of cases). The mean level of B_12_ vitamin was 378.5 ± 151.8 ng/ml with levels under 200 ng/L in 8 participants (14.0%); vitamin D mean value was 26.8 ± 9.3 ng/ml. No patients had an alteration in all of the three parameters. The folate levels were normal in all patients, with a mean value of 9.8 ± 13.7 ng/ml.

### Neurocognitive examination

All cognitive impaired patients showed an ANI profile type, without significant interference in the everyday life (IADL≥7). By analyzing the proportion of impairments, which scored below the normal cut-off, for each single task, median values did not significantly differ with the normal parameter, but 42 patients (73.7%) showed three or more pathologic tasks with ANI: 33 patients had more than 2 pathologic tests (57.9%), 24 patients had more than three pathologic tests (43.6%) and 16 patients had more than 4 pathologic tests (29.1%). The overall tests assessment showed that 21.1% of patients had pathological result in the battery exploring Memory tests and 19.3% of them showed problems in long time-memory; 66.7% of patients showed a pathological test of attention and working memory, 57.9% was pathologic on executive functions, 31.6% had low points on speed of psychomotor processing. For 12 patients (21.1%) there was a significant pathologic result in Zung Depression scores. Raw scores for each task and comparison cut-off are illustrated in Table 2.

**Table 2:** Cognitive test performances

	Median (IQR)	Normality Range/Cut-off	Pathologic Test, n (%)
MEMORY			12 (21.1)*
Rey BT	39.0 (34.0–44.0)	> 28.53	9 (15.8)
Rey LT	8.0 (6.0–10.0)	> 4.69	11 (19.3)
ATTENTION AND WORKING MEMORY			38 (66.7)*
Verbal Span forward	5.0 (4.0–6.0)	5.00–9.00	18 (31.6)
Spatial Span forward	3.0 (3.0–5.0)	5.00–9.00	37 (64.9)
Double barrage	1.0 (1.0–1.0)	> 0.92	6 (10.5)
EXECUTIVE FUNCTIONS			33 (57.9)*
Stroop E	1.0 (0–2.0)	< 4.24	8 (14.0)
Stroop T	19.0 (14.0–28.0)	< 36.91	10 (17.5)
Trail making test B - T	149.0 (112.0–220.0)	111.29″-248.89″	10 (17.5)
Trail making test B - E	1.0 (0–2.0)	−0.21-2.39	6 (10.5)
Drawings	5.0 (3.0–6.0)	3.85–6.15	16 (28.1)
Wais Digit span	33.0 (23.0–45.0)	> 6.00	14 (24.6)
LANGUAGE			5 (8.8)*
Verbal fluency test	33.0 (27.0–41.0)	> 17.35	5 (8.8)
SPEED OF PSYCHOMOTOR PROCESSING			18 (31.6)*
Pegboard dom	72.0 (62.0–82.0)	55.94–74.32	18 (31.6)
Pegboard non dom	75.0 (68.0–85.0)	59.68–80.30	10 (17.5)
DEPRESSION			12 (21.1)*
Zung depression score	38.0 (31.0–46.0)	< 50.00	12 (21.1)
DAILY LIFE ACTIVITY			–
IADL	8.0 (8.0–8.0)	< 7.00	–

*Patients with at least one pathologic test in the relative domain. *IQR* Interquartile Range

### Clinical variables and neurocognitive functions

**Fig. 1:**
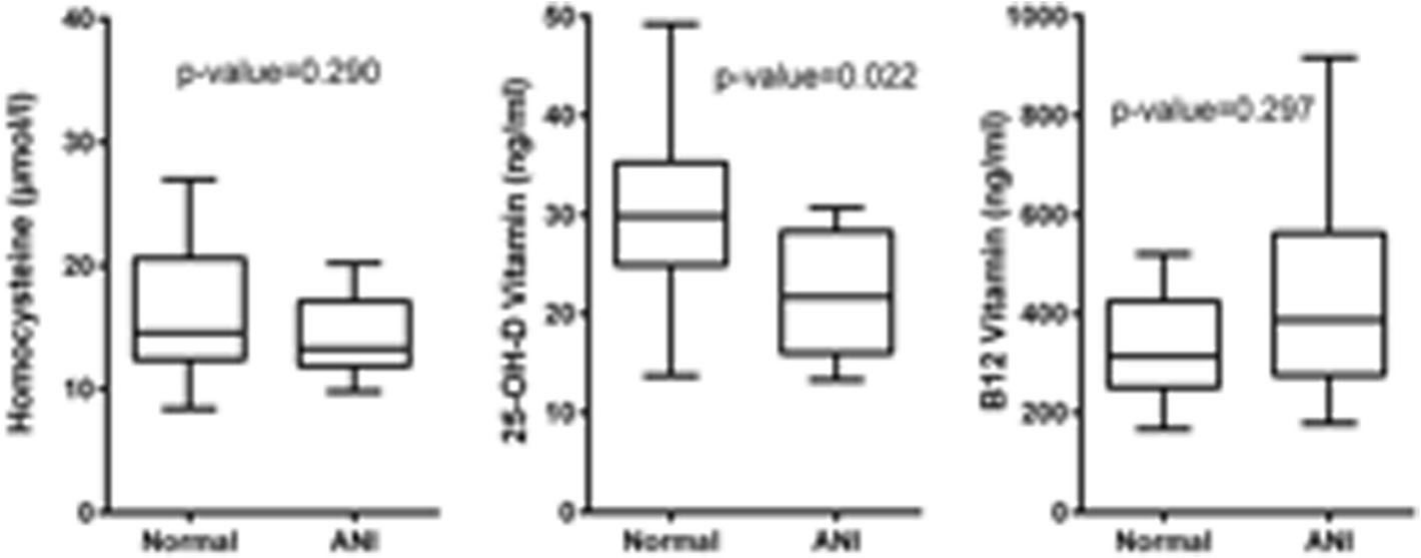
Levels of Homocysteine, D and B_12_ Vitamin between patients with less and more than 3 pathological cognitive tests

Abnormal homocysteine levels were associated with worsened performance in verbal fluency (*p* = 0.003) and worse executive functions (Stroop E test *p* = 0.040). 25-OH D hypovitaminosis was associated with worse performances in executive functions in three different test: Stroop E (*p* = 0.049), Trail B (*p* = 0.035) and Wais Digit Span (*p* = 0.042). Pathological levels of B_12_ vitamin were also associated with worse performances in executive functions (Trail B Test and Wais Digit Span respectively *p* = 0.002 and 0.029) and with lower speed of psychomotor processing (Peg Board Test on dominant hand, *p* = 0.014) (Table 3). No significant relations were found among pharmacological therapy, HBV/HCV co-infections and vitamins levels. The group that received an ANI diagnosis (over 3 pathologic neurocognitive tests) showed lower levels of 25-OH D vitamin compared with the patients that had less than 3 pathologic tests (*p* = 0.022) (Fig. 1). No difference in homocysteine levels and B_12_ vitamin levels were found between patients with less and more than 3 pathological cognitive tests.

**Table 3:** Differences in cognitive test among patients with or without pathologic values of Homocysteine, 25-OH D Vitamin and B_12_ Vitamin

Cognitive test	Homocysteine		*p-value* ^*a*^	25-OH D Vitamin		*p-value* ^*a*^	B-12 Vitamin		
	Non-Pathologic (*n* = 17)	Pathologic (*n* = 40)		Non-Pathologic (*n* = 49)	Pathologic (*n* = 8)		Non-Pathologic (n = 49)	Pathologic (n = 8)	*p-value* ^*a*^
Rey word BT	38.0 (31.0–43.3)	39.0 (32.5–44.3)	*0.897*	39.5 (34.0–44.3)	31.0 (22.5–38.8)	*0.144*	39.5 (33.5–45.5)	39.0 (23.0–40.5)	*0.395*
Rey word LT	7.0 (4.0–10.0)	8.0 (6.0–9.8)	*0.401*	7.0 (5.0–9.8)	5.5 (4.0–7.8)	*0.195*	7.5 (5.0–10.0)	7.0 (4.0–9.0)	*0.421*
Verbal Span Forward	5.0 (3.0–6.0)	5.0 (4.0–6.0)	*0.615*	5.0 (4.0–6.0)	5.0 (5.0–5.8)	*0.589*	5.0 (4.0–6.0)	5.0 (2.5–5.5)	*0.873*
Spatial Span Forward	4.0 (3.0–5.0)	3.5 (3.0–5.0)	*0.662*	4.0 (3.0–5.0)	4.0 (3.0–5.0)	*0.874*	4.0 (3.0–5.0)	5.0 (2.5–5.5)	*0.567*
Stroop E Test	2.0 (1.8–3.5)	1.0 (0–2.0)	***0.040***	1.0 (0–2.0)	3.0 (2.0–10.8)	***0.049***	1.5 (0–2.0)	4.0 (0.5–9.5)	*0.155*
Stroop T Test	20.0 (13.5–38.5)	17.0 (12.3–26.0)	*0.565*	17.0 (13.3–26.0)	24.0 (14.5–39.5)	*0.065*	16.0 (12.8–26.3)	26.0 (17.0–50.0)	*0.185*
Trail B T	154.0 (107.0–236.3)	137.5 (92.3–213.0)	*0.660*	131.5 (96.0–195.3)	281.0 (152.3–403.8)	***0.035***	133.5 (93.8–198.8)	338.0 (186.5–387.5)	***0.002***
Trail B E	1.0 (0–1.8)	0 (0–2.0)	*0.298*	0 (0–2.0)	1.5 (0.3–3.5)	*0.408*	0 (0–1.3)	2.0 (0–4.0)	*0.257*
Drawings	5.5 (4.0–6.0)	4.5 (2.3–6.0)	*0.170*	4.5 (3.0–6.0)	2.0 (0–5.5)	*0.157*	5.0 (4.0–6.0)	2.0 (0.5–4.0)	*0.054*
Verbal Fluency	24.0 (17.0–33.3)	36.5 (29.3–48.0)	***0.003***	34.5 (29.0–41.5)	23.0 (16.0–42.0)	*0.629*	34.5 (27–47.3)	24.0 (17.0–39.5)	*0.237*
Wais Digit Span	29.5 (19.3–49.3)	33.5 (21.5–44.0)	*0.909*	32.0 (23.0–44.0)	12.5 (6.8–29.5)	***0.042***	33.5 (22.5–46.3)	9.0 (6.5–32.0)	***0.029***
Double Barrage	12.0 (9.0–12.3)	11.0 (10.0–12.8)	*0.836*	11.5 (10.3–12.0)	10.5 (9.3–12.5)	*0.195*	11.0 (10.0–12.0)	13.0 (9.5–13.0)	*0.395*
Pegboard Dominant	76.0 (66.5–79.8)	73.5 (64.5–87.8)	*0.945*	75.0 (67.0–80.0)	97.5 (71.3–145.5)	*0.376*	72.0 (64.8–80.0)	100.0 (81.0–131.0)	***0.014***
Pegboard Non-Dominant	75.5 (69.0–86.8)	77.0 (68.3–90.5)	*0.982*	77.5 (69.3–85.8)	96.5 (78.5–146.8)	*0.075*	75.0 (67.5–87.5)	83.0 (76.5–131.5)	*0.095*
Zung Depression Score	43.5 (33.5–53.0)	34.5 (29.3–44.8)	*0.060*	36.5 (29.3–48.0)	45.5 (41.3–56.5)	*0.755*	38.0 (30.8–46.0)	44.0 (31.0–53.0)	*0.506*

^a^Mann-Whitney U test

bolded *p*-value were significant after FDR correction

Logistic regression analysis confirmed the protective activity of 25-OH D hypovitaminosis on the risk of ANI (OR = 0.891; *p* = 0.042), as showed in Table 4.

**Table 4:** Results of logistic regression analyses performed to estimate the risk of ANI

	Odds Ratio (95%CI)	*p-value*
25-0H D Vitamin (ng/ml)	0.891 (0.798–0.996)	***0.042***
Homocysteine (μmol/l)	0.922 (0.813–1.046)	*0.207*
B_12_ Vitamin (ng/ml)	1.003 (0.998–1.008)	*0.214*

*CI* Confidence Interval

## Discussion

In this study, a population with high rates of altered metabolic parameters was examined, such as: homocysteine, vitamin B_12_, and vitamin D; more than half of the patients showed high levels of homocysteine and B_12_ hypovitaminosis, while almost all showed a D hypovitaminosis.

B_12_ Vitamin and folates are closely linked to the metabolism of homocysteine, a non-essential amino acid of sulphuric nature. According to Hooshmand et al., high levels of this amino acid, along with low levels of vitamin and folate, are often associated with a variety of diseases, including cardiovascular and cerebral illnesses, and can also damage the brain structure causing brain atrophy and progression of white substance lesions [4]. These brain atrophy related factors are significant given their modifiability and this can be important for the prevention of neurocognitive disorders [19].

The role of homocysteine in the pathological processes of the CNS was described around the 60s of the last century, with the demonstration that an error in this amino acid metabolism caused mental retardation in pediatric subjects. About ten years later, homocysteine showed to have deleterious effects also at the vascular level and that it could become a marker of atherosclerosis [20].

Association between elevated homocysteine plasma levels and cognitive impairment in HIV-negative individuals has been the topic of many studies. Data suggest an association between elevated levels of homocysteine and cognitive impairment such as Alzheimer’s [21]. There is anyway a lack of data about homocysteine and vitamins levels in the context of CNS injury and neurocognition in the case of HIV infected individuals [4, 13]. Among the few studies in literature, we can mention one by Gisslén et al. this group found a correlation between plasma homocysteine levels and neurofilament light protein, a marker of neuronal injury, in cerebrospinal fluid of HIV-positive patients, pointing to a possible role of homocysteine in neuronal injury in HIV [13].

In our study, high homocysteine plasma levels seem to be associated with a worsening in the speed of executive functions and with a less fluent language. Vitamin D levels were associated with altered tests for the evaluation of executive functions and vitamin B_12_ levels were correlated with a worse performance in executive functions and speed of psychomotor processing.

Previous paper reported a possible correlation between hyperhomocysteinemia and cardiovascular risk in HIV-positive patients treated with Protease Inhibitors (IP) and have found a statistical correlation between hyperhomocysteinemia and low levels of folate and B_12_ [22]. Other studies show that cART has a low prevalence of B_12_ hypovitaminosis [23], and that hyperhomocysteinemia correlates with serum HIV-RNA levels in patients not on cART [13]. Dysfunctions in acquisition and processing speeds, visual-space abnormalities and problem solving have been reported in patients without cART, while patients with ANI have increased plasma homocysteine levels resulting in axonal injury. Not less surprising is the observation that MTHFR (677C → T) polymorphisms, altered levels of vitamin B_12_, and other metabolic markers (neopterin in the brain, NO, etc.) seem to be dose-dependent, depression risk factors [24].

In this analysis all patients were treated with cART, had undetectable viral load and showed that pathologic levels of homocysteine, B_12_ and D vitamins were associated to worsened performances in the tests for executive functions.

There is some evidence that D vitamin deficiency is associated with an increased risk for vascular diseases, cognitive decline and also with increased general mortality [25–27] and with more brain tissue loss [28]. In this study participants exhibited a statistically significant correlation between D vitamins and neurocognitive tests scoring. In fact ANI was the condition showing the strongest association with D hypovitaminosis.

There are some limitations of this study. The study group comprised a small number of participants and therefore results should be interpreted with caution. In addition, the study could be limited by multiple comparison analyses, despite FDR correction was performed. These conclusions were associated with Italian people because participants analysed were only Italian for the neurocognitive test requirements. This was a transversal study of closely followed up patients with no missing data but with no control group involved.

## Conclusions

Our findings show that serum homocysteine, B_12_ vitamin and D vitamin levels are correlated to neurocognitive performances. Evidence of the alteration of these parameters could facilitate the early identification of a neurocognitive impairment. There are some evidences that early detection and management of these laboratory parameters may contribute both to primary and to secondary prevention for neurologic involution.

It could be appropriate to assess in large multicenter intervention studies if the reduction of homocysteine levels and the increase in D and B_12_ vitamins levels are linked to significant neurocognitive performances improvement.

## Abbreviations

cART: Combination antiretroviral therapy
HAND: HIV-associated neurocognitive disorders
ANI: Asymptomatic Neurocognitive Impairment
IADL: Instrumental Activities of Daily Living
HDL: High-density lipoprotein-cholesterol
LDL: Low-density lipoprotein
SD: Standard deviation
IQR: Median and interquartile range
OR: Odds ratio
BMI: Body mass index
IP: Protease Inhibitors.
